# LATS2 is De-methylated and Overexpressed in Nasopharyngeal Carcinoma and Predicts Poor Prognosis

**DOI:** 10.1186/1471-2407-10-538

**Published:** 2010-10-08

**Authors:** Yan Zhang, Chun-Fang Hu, Jing Chen, Li-Xu Yan, Yi-Xin Zeng, Jian-Yong Shao

**Affiliations:** 1 State Key Laboratory of Oncology in Southern China, Sun Yat-Sen University Cancer Center, Guangzhou, China; 2 Department of Pathology, Sun Yat-Sen University Cancer Center, Guangzhou, China; 3 Institute for Cancer Studies, University of Birmingham, Birmingham, UK; 4 Department of Experiment Research, Sun Yat-Sen University Cancer Center, Guangzhou, China

## Abstract

**Background:**

LATS2, which encodes a novel serine/threonine kinase, is known to be important in centrosome duplication and in the maintenance of genomic stability. Recently, a potential role for LATS2 in cancer has been reported. In breast cancer and acute lymphoblastic leukemia (ALL), LATS2 mRNA is downregulated and has been suggested to be a tumor suppressor. However, the role of LATS2 in nasopharyngeal carcinoma has not been investigated. In this study, we aimed to investigate the expression pattern of LATS2 and its clinicopathological involvement in nasopharyngeal carcinoma to understand its effect on cell survival.

**Methods:**

Using quantitative real time PCR and immunoblotting, the expression of LATS2 was detected in nasopharyngeal carcinoma cell lines and in the immortalized nasopharyngeal epithelial cell line NP69. Using immunohistochemistry, we analyzed LATS2 protein expression in 220 nasopharyngeal carcinoma cases. The association of LATS2 protein expression with the clinicopathological characteristics and the prognosis of nasopharyngeal carcinoma were subsequently assessed. Using methylation specific PCR, we detected the methylation status of the LATS2 promoter. RNA interference was performed by transfecting siRNA to specifically knock down LATS2 expression in 5-8F and CNE2.

**Results:**

LATS2 protein was detected in 178 of 220 (80.91%) cases of nasopharyngeal carcinoma. LATS2 overexpression was a significant, independent prognosis predictor (*P *= 0.037) in nasopharyngeal carcinoma patients. Methylation specific PCR revealed that 36.7% (11/30) of nasopharyngeal carcinoma tissues and all of the chronic nasopharyngeal inflammation samples were methylated. Functional studies showed that the suppression of LATS2 expression in nasopharyngeal carcinoma (5-8F and CNE2) cell lines by using specific small interfering (siRNA) resulted in the inhibition of growth, induction of apoptosis and S-phase cell cycle increase. Overexpression of LATS2 in NP69 stimulated cell proliferation.

**Conclusions:**

Our results indicate that LATS2 might play a role in the tumorigenesis of nasopharyngeal carcinoma by promoting the growth of nasopharyngeal carcinoma cells. Transfection with specific siRNA might be feasible for the inhibition of growth, induction of apoptosis and S phase increase in nasopharyngeal carcinoma.

## Background

Nasopharyngeal carcinoma (NPC) is endemic to certain areas of Southern China, South-Asia and North Africa. In Southern China in the Guandong and Guangxi provinces, the incidence rate of NPC is up to 25-40 per 100,000 person-years [1,2]. A dominant clinicopathological characteristic of NPC is the involvement of cervical lymph nodes and distant metastasis, compared with other head and neck carcinomas. Although the current treatment regimen for NPC is fractionated radiotherapy, adjunctive chemotherapy has shown promise by improving tumor control and survival in advanced nasopharyngeal carcinoma [3-5]. NPC is associated with a high rate of treatment failure because of local recurrence and distant metastasis [6-8]. Published reports indicate that the etiologic factors associated with NPC are genetic susceptibility [9], EBV infection [10], and other environmental factors [11,12]. However, the precise genetic alterations responsible for NPC development, progression and metastasis are unknown. Therefore, it is of great clinical value to find factors for early diagnosis and prognosis prediction, as well as novel therapeutic strategies, and it is critical to further understand the molecular mechanism of NPC.

LATS2 (Large Tumor Suppressor homolog 2), also known as Kpm, is one of the two human homologues of *Drosophila *wts, which is a component of the Hippo pathway. This pathway is now known to control organ size by modulating cell growth, proliferation, and apoptosis [13,14]. Recent work suggests that LATS2 regulates both growth and death of cardiac myocytes, and that it is a negative regulator of myocyte size in the heart [15]. LATS2 inhibits cell proliferation by inducing G2/M arrest through the inhibition of cdc2 kinase activity [16], or by blocking G1/S transition through the down-regulation of cyclin E/CDK2 kinase activity [16,17]. Ectopic expression of LATS2 in human lung cancer cells induces apoptosis via down-regulation of apoptotic inhibitors such as Bcl-2 and Bcl-xL [18]. LATS2 binds to Mdm2 and inhibits its E3 ubiquitin ligase activity, resulting in the stabilization of p53 in nocodazole treated cells, while p53 rapidly and selectively up-regulates LATS2 expression in G2/M cells. This process is therefore a positive feedback loop between p53 and LATS2 [19]. LATS2 is also required for embryonic development, proliferation control and genomic integrity [20,21]. LATS2 -/- MEFs display defects in contact inhibition of growth, cytokinesis failure, centrosome amplification, multipolar mitotic spindles and genomic instability. However, disruption of LATS2 results in embryonic lethality. In LATS2-/- embryos, the development of the nervous system was severely impaired, and deficiency in LATS2 results in growth arrest and apoptosis.

LATS2 has not been widely studied in the field of cancer. The aim of the present study is thus to investigate the expression pattern of LATS2 and its clinicopathological implication for NPC, and to further understand its effect on cell survival. We showed that overexpression and de-methylation of LATS2 occurred very frequently in NPC tissues, and overexpression of LATS2 predicted poor prognosis of NPC patients. Furthermore, suppression of LATS2 expression resulted in growth inhibition, apoptosis and S-phase increase. Overexpression of LATS2 stimulated cell growth. These results suggest that LATS2 might play a role in the tumorigenesis of NPC and might be a potential therapeutic target in NPC treatment.

## Methods

### Patients and clinical tissue samples

For this retrospective study, archival formalin-fixed, paraffin-embedded specimens from 220 primary NPC patients during 1992 ~ 2002 in Sun Yat-Sen University Cancer Center (Guangzhou, China) were collected. All the NPC samples in our study were obtained before treatment with standard curative radiotherapy with or without chemotherapy. Patients included 166 males and 54 females with ages ranging from 14 to 86 years (median age 46 years). The disease stages of all the patients were classified according to the 1992 NPC staging system of China as mentioned before [22]; 6 patients were in stage I, 48 patients in stage II, 103 patients in stage III and 63 patients in stage IV. Among 220 patients, 58 patients were diagnosed as differentiated non-keratinising carcinoma (WHO type II) and 162 patients were diagnosed as undifferentiated carcinoma (WHO type III). All patients were treated with standard curative radiotherapy with or without chemotherapy. Information on date of death and cancer as the cause of death was obtained from the Sun Yat-Sen University Cancer Center Registry. Patients who died from diseases other than NPC or from unexpected events were excluded from the study. The median follow-up period was 44 months (range, 2-120 months). A total of 66 (30.00%) patients died during follow-up and 29 (13.18%) patients experienced metastasis. The study was approved by the Research Ethics Committee of Sun Yat-Sen University Cancer Center (Reference number: YP-2009169)

### Tissue microarray construction

Paraffin-embedded specimens from the previously constructed tissue microarray (TMA), and the procedures for the TMA construction were described previously [23,24]

### Immunohistochemistry

Immunohistochemistry was performed to examine LATS2 and p-YAP (Phospho-YAP [Ser127]) expression in nasopharyngeal carcinoma tissue specimens. A primary antibody against LATS2 (1:400 dilution, sc-23065, Santa Cruz, USA) and p-YAP (1:200, #4911, Cell Signaling Technology) were used in this study. The staining protocol used was as previously described. The immunohistochemistry results were evaluated and scored independently by two pathologists without knowledge of the clinicopathological outcomes of the patients. A semiquantitative estimation was made using a composite score obtained by adding the values of the staining intensity and the relative abundance of the positive cells. The intensity was graded as 0 (negative), 1 (weakly positive), 2 (moderately positive) and 3 (strongly positive). The abundance of the positive cells was graded from 0 to 4 (0, < 5% positive cells; 1, 5-25%; 2, 26-50%; 3, 51-75%; 4, 76-100%). A composite score greater than or equal to the median value was considered high expression, and composite scores below the mean value were considered low expression. To ensure the specificity of LATS2 antibody, non-immunized goat IgG antibody was used as a negative control.

Immunohistochemistry of cultured cells was performed as follow. Cultured cells were harvested and washed thrice with PBS. Then resuspended with PBS and centrifuged on the slides by cytospin machine Rotofix 32 (Hettich, Germany). The cells on the slides were fixed with 4% paraformaldehyde for 30 min, permeabilization steps followed, cover cells with 0.25% Triton X-100 for 15 min. Immunohistochemistry was performed to examine LATS2 expression in 5-8F cells or transfected cells.

### Methylation specific PCR

Genomic DNA was isolated from microdissected NPC samples and NPC cell lines, and then modified by sodium bisulfite [25]. The methylation status of the promoter regions of LATS2 was previously reported [26]. Modified DNA was prepared for PCR with methylation-specific primers (5'-ATT TCG GTT TAT TGT AAT TTT C-3' and 5'-AAC CAA CAT AAT AAA ACC CCG-3') and unmethylation-specific primers (5'-TTT GTT TTT TGG GTT TAA GT-3' and 5'-CCA ACA TAA TAA AAC CCC A-3') [26]. PCR was carried out in 25 μL containing 2 μL modified DNA, 2× GC buffer 12.5 μL (Takara, Japan), 4×10 mmol/L dNTP 1 μL, 10 mmol/L of each primer 1 μL, and 5 U/μL Takara LA Taq DNA polymerase 0.25 μL (Takara, Japan). Further amplification conditions were as follows: after initial denaturation at 94°C for 3 minutes, 40 cycles at 94°C for 30 seconds, 50°C for 30 seconds, and 72°C for 30 seconds, followed by a 6-minute extension at 72°C. PCR products were separated on 2.5% agarose gels and visualized under UV illumination with ethidium bromide staining.

### Cell lines and cell culture

The three NPC cell lines CNE1, CNE2, and 5-8F were cultured in RPMI 1640 (Gibco, USA) with 10% fetal bovine serum (Gibco, USA). The human NPC cell line C666-1 was cultured in RPMI 1640 medium (GIBCO, USA) containing 15% FCS. The immortalized nasopharyngeal epithelial cell line NP69 was cultured in keratinocyte serum-free medium (Invitrogen) supplemented with 5% FCS, 25 μg/ml bovine pituitary extract, and 0.2 ng/ml recombinant epidermal growth factor, as suggested by the manufacturer. All the cell lines were grown in a humidified incubator at 37°C with 5% CO_2_.

### Real time PCR assays

Total RNA was extracted from the NPC cell lines CNE1, CNE2, 5-8F, C666-1 and an immortalized nasopharyngeal epithelial cell line, NP69, as well as three NPC biopsies and the paired normal tissues by using TRIzol reagent (Invitrogen). After reverse transcription of the total RNA, the first-strand cDNA was then used as template for detection of LATS2 expression by using quantitative real time PCR (QT-PCR) with the SYBR Green I chemistry (ABI Inc., USA). GAPDH was used as control. The primers of LATS2 were (Forward: 5'-TGG CAC CTA CTC CCA CAG-3' and Reverse: 5'-CCA AGG GCT TTC TTC ATC T-3'), GAPDH (Forward: 5'-CCA CCC ATG GCA AAT TCC ATG GCA-3' and Reverse: 5'-TCT AGA CGG CAG GTC AGG TCC ACC-3'). Thermal cycle conditions were: 1 cycle of pre-incubation at 95°C for 5 minutes (segment 1); 40 cycles of amplification at 95°C for 30 seconds, 58°C (LATS2) or 57°C (GAPDH) 45 seconds and 72°C for 45 seconds (segment 2); and melting temperature curve analysis at 95°C for 30 seconds, 60°C for 30 seconds and 95°C for 30 seconds (segment 3). The relative expression level was determined as 2^-ΔΔCt^. Data are presented as the expression level relative to the calibrator (NP69 cells), with the standard error of the mean of triplicate measures for each test sample.

### Cell transfection

Three siRNAs (GenePharma, China) designed against LATS2 (GenBank accession no. NM_014572) were included in this study. One control siRNA (GenePharma, China) exhibiting no significant sequence similarity to human, mouse or rat gene sequence served as a negative control. The sequences for LATS2 specific siRNAs and control siRNA are: siRNA1 (GCC UCA ACG UGG ACC UGU ATT), siRNA2 (GGA CAA AGG CGG AAA GGA UTT), siRNA3 (GGA CCU UCA CUG CAU UAA ATT) and control siRNA (UUC UCC GAA CGU GUC ACG UTT).

The plasmids for pCMVmyc-LATS2, described elsewhere [27], were a gift of Dr Hiroshi Nojima (Osaka University). Full LATS2 cDNA was released from the pCMVmyc-LATS2 plasmid by BamHI and XhoI cleavage and recloned into the pcDNA3 vector to produce pcDNA3-LATS2.

Transfection of siRNA and plasmid was performed with lipofectamine 2000 transfection reagent (Invitrogen, USA) following the manufacturer's protocol. Transfected cells were grown at 37°C for 6 h, followed by incubation with complete medium.

### Cell growth assay

For cell growth assay, at 24 h after the treatment, the cells of each group were reseeded in 24-well plates at a density of 0.8 × 10^4 ^cells per well, then harvested at 24-h intervals up to 6 days. The number of living cells was counted with trypan blue exclusion.

### MTT assay

For cell proliferation assay, at 24 h after transfection, the cells of each group were reseeded in 96-well plates at a density of 2 × 10^3 ^cells/well and incubated overnight in 100 μL of the culture medium. Cells without any treatment were used as controls. A volume of 20 μL of 5 mg/mL MTT (Sigma, USA) labeling reagent was added to the wells, and cells were incubated at 37°C for 4 h. The supernatant was removed, then 150 μL dimethyl sulfoxide (DMSO, Sigma, USA) was added to the wells. After the plate was incubated at 37°C for 15 min, the absorbance was measured with a microplate reader (SpectraMax M5, Molecular Devices USA) at a wavelength of 570 nm.

### Apoptosis assay

Apoptotic cell death was assessed by Annexin-V FITC and propidium iodide (PI) double staining to discriminate apoptotic cells from live cells. In brief, after the treatment, cells of each group were stained according to the instructions of the Annexin-V FITC Apoptosis Kit (Invitrogen, USA), and analyzed by a flow cytometer (EPICS ELITE, COULTER, USA). The data were presented as dot plots showing fluorescence intensity of Annexin-V FITC and PI.

### Cell cycle analysis

After the treatment, cells were trypsinized and concentrated by mild centrifugation. The cell pellet was resuspended, washed three times with cold PBS, and fixed with cold 75% ethanol at 4°C overnight. Then, the cells were washed once with PBS and digested with RNase at 37°C for 30 min. Nuclei of cells were stained with propidium iodide at room temperature for 30 min. A total of 10,000 nuclei were examined in a flow cytometer and DNA histograms were analyzed by Modifit software (Verity Software House, USA).

### Immunoblot analysis

Cells were harvested and lysed with RIPA buffer (Upstate, USA). Equal amounts of denatured protein sample were separated by SDS-PAGE and then transferred electrophoretically to PVDF membranes (Amersham, UK) for immunoblot analysis. Antibodies used for immunoblot analysis were against LATS2 (1:500 dilution, sc-23065, Santa Cruz, USA), an anti-GAPDH antibody (1:3,000 dilution, sc-32233, Santa Cruz, USA) was used as loading control. All protein bands were detected using an enhanced chemiluminescent (ECL) Western blot Kit (Amersham, UK).

### Statistical analysis

Data were analyzed using SPSS13.0 software. Associations between the expression of LATS2 and clinicopathological parameters were assessed using a Chi-Square test. Survival curves were plotted by Kaplan-Meier analysis and compared by the log-rank test. Cox regression analysis was performed to assess the significance of various variables for survival. Results for the cell growth assay were expressed as mean ± S.D, and T-test was used to determine the significance of differences in multiple comparisons. *P *< 0.05 was considered statistically significant.

## Results

### Expression of LATS2 and its association with clinicopathological parameters in NPC

**Figure 1:**
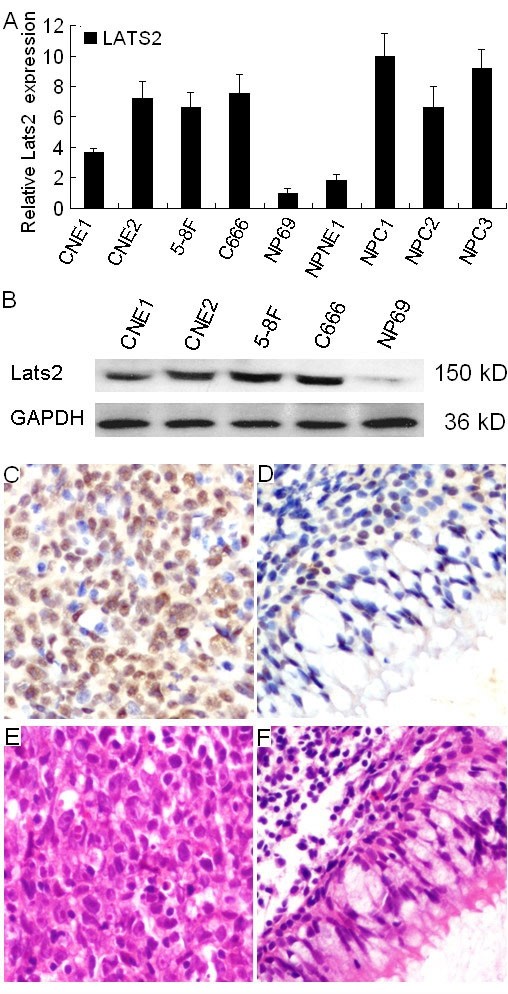
**Expression of LATS2 in NPC cell lines and NPC tumor tissues**. A, The expression levels of LATS2 transcripts in the NPC cell lines CNE1, CNE2, 5-8F and C666-1, and in immortalized primary nasopharyngeal epithelial NP69 cells, as well as in three NPC biopsies and one paired normal epithelium were evaluated by quantitative real-time PCR. The NPC cell lines CNE1, CNE2, 5-8F and C666-1, as well as NPC tumors (NPC1, NPC2 and NPC3) show a higher LATS2 mRNA expression level than immortalized primary nasopharyngeal epithelial NP69 cells and normal nasopharynx epithelium (NPNE1). B, The LATS2 protein expression level was detected by western blot in the NPC cell lines CNE1, CNE2, 5-8F, C666-1 cells and in NP69 cells. C, LATS2 (IHC, × 200) staining revealed that overexpression of LATS2 was observed in the nuclear and cytoplasm of carcinoma cells. D, Weak staining of LATS2 was observed in adjacent normal nasopharyngeal epithelial cells. H&E staining of NPC carcinoma cells (E) and adjacent normal nasopharyngeal epithelial cells (F).

To determine LATS2 expression in NPC, quantitative real-time PCR was performed to evaluate the expression levels of LATS2 transcripts in the NPC cell lines CNE1, CNE2, 5-8F and C666-1, in an immortalized primary nasopharyngeal epithelial cell line NP69, as well as in three NPC biopsies and their paired normal tissues. Compared to NP69 cells, overexpression of LATS2 mRNA was observed in the NPC cell lines CNE1, CNE2, 5-8F and C666-1. The expression levels of LATS2 mRNA were also significantly up-regulated in three NPC tumor tissues, compared to the paired normal tissues (Fig. 1A). Western-blot analysis also revealed overexpression of LATS2 protein in CNE1, CNE2, 5-8F and C666-1 cells, compared to NP69 cells (Fig. 1B)

Expression of LATS2 protein was determined by immunohistochemistry in 220 NPC tissues. LATS2 immunostaining revealed that overexpression of LATS2 was observed in the nuclear and cytoplasm of NPC carcinoma cells (Fig. 1C) and 5-8F cell line (Additional file 1). LATS2 was expressed weakly in normal nasopharyngeal epithelium (Fig. 1D). However, no immunostaining signal was detected in NPC tumor tissue and 5-8F cell line incubated with a non-immunized goat IgG antibody (Additional file 1). LATS2 protein was detected in 178 of 220 (80.91%) cases of NPC. No significant association between LATS2 expression and age, gender, clinical stage, histological type (WHO), recurrence or distant metastasis of NPC was observed (Table 1).

**Table 1:** Correlation between LATS2 expression and clinicopathological parameters of NPC

Parameters	Cases (n = 220)	LATS2 expression		*P *value
			
		Low expression (n = 109)	High expression (n = 111)	
Age				
< 46	104	56	48	0.227
≥46	116	53	63	
Gender				
Male	166	77	89	0.100
Female	54	32	22	
T stage				
T1+T2	90	49	41	0.227
T3+T4	130	60	70	
N stage				
N0	58	29	29	0.936
N1+N2+N3	162	80	82	
clinical stage				
I + II	54	29	25	0.482
III + IV	166	80	86	
Histological type (WHO)				
II	58	28	30	0.882
III	162	81	81	
Recurrence				
Yes	54	26	28	0.813
No	166	83	83	
Metastasis				
Yes	29	14	15	0.883
No	191	95	96	

Median age is 46 years.

### Association between LATS2 expression and survival of NPC patients

**Figure 2:**
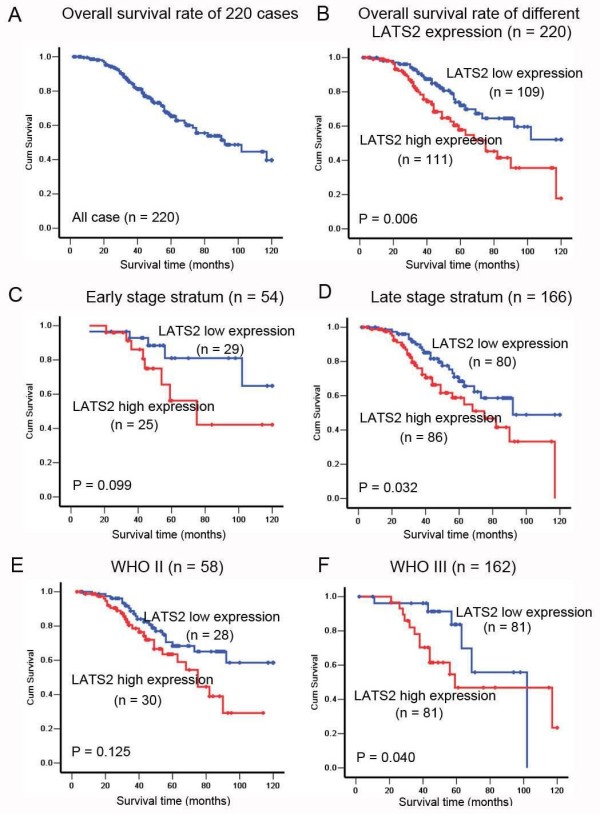
**Kaplan-Meier survival curves of NPC patients**. A, The five-year overall survival rate of 220 NPC patients was 66.18%. B, The five-year overall survival rates were 73.96% and 57.23%, in NPC patients whose tumors showed low levels of LATS2 expression (n = 109) and high levels of LATS2 expression (n = 111), respectively; there was a significant difference in the overall survival rate between the two groups (*P *= 0.006). C, No significant differences in five-year survival rates were found between low levels of LATS2 expression (n = 29) and high levels of LATS2 expression (n = 25) in NPC patients with early stage disease (stage I - II, *P *= 0.099). D, The five-year overall survival rates were 70.57% and 57.59% in patients with late stage disease (stage III - IV) whose tumors showed low levels of LATS2 expression (n = 80) and high levels of LATS2 expression (n = 86), respectively; there was a significant difference in the overall survival rate between the two groups (*P *= 0.032). E, No differences were found in overall survival between low levels of LATS2 expression (n = 28) and high levels of LATS2 expression (n = 30) in NPC patients with differentiated non-keratinising carcinoma (WHO type II, *P *= 0.125). F, The five-year overall survival rates were 70.91% and 62.23% in patients with undifferentiated carcinoma (WHO type III) whose tumors showed low levels of LATS2 expression (n = 81) and high levels of LATS2 expression (n = 81), respectively; there was a significant difference in the overall survival rate between the two groups (*P *= 0.040).

The 5-year overall survival rate of the cohort of 220 NPC patients was 66.18% (Fig. 2A). Kaplan-Meier survival analysis revealed that NPC patients with high LATS2 expression had a significantly poor prognosis compared to those with low LATS2 expression. The 5-year survival rates in patients with low LATS2 expression (n = 109) and high LATS2 expression (n = 111) were 73.96% and 57.23%, respectively; there was a significant difference between the two groups (log-rank test, X^2 ^= 7.660, *P *= 0.006, Fig. 2B). When the patients were stratified by clinical stage, LATS2 expression was not related to the patients survival in the early stage (stage I - II) cases, (log-rank test, X^2 ^= 2.728, *P *= 0.099, Fig. 2C). Within the late stage cases (stage III - IV), Kaplan-Meier survival estimates revealed that patients with high LATS2 expression (n = 86) had inferior survival to those with low LATS2 expression (n = 80, log-rank test, X^2 ^= 4.589, *P *= 0.032, Fig. 2D). When the patients were stratified by histological type (WHO), LATS2 expression was not related to patient survival in the type II cases, (log-rank test, X^2 ^= 2.351, *P *= 0.125, Fig. 2E). Within the type III cases, Kaplan-Meier survival estimates revealed that patients with high LATS2 expression (n = 81) had inferior survival to those with low LATS2 expression (n = 81, log-rank test, X^2 ^= 4.207, *P *= 0.040, Fig. 2F).

Univariate Cox proportional hazard regression analysis revealed that high LATS2 expression was a significant predictive factor for poor prognosis of patients with NPC (Hazard ratio (HR) = 1.978, 95% confidence interval [CI]: 1.207-3.241, *P *= 0.007,). Other clinicopathologic parameters, including gender (HR = 0.380, 95% CI: 0.193-0.746, *P *= 0.005), metastasis (HR = 2.578, 95% CI: 1.424-4.665, *P *= 0.002) were also found to be prognostic predictors of overall survival in NPC patients (Table 2). Multivariate Cox proportional hazards regression analysis indicated that LATS2 expression (HR = 1.714, 95% CI: 1.033 - 2.843, *P *= 0.037,), gender (HR = 0.437, 95% CI: 0.219 - 0.874, *P *= 0.019) and metastasis ((HR = 2.677, 95% CI: 1.424 - 5.031, *P *= 0.002) were predictors for death (Table 2).

**Table 2:** Univariate and multivariate Cox regression analysis of different prognostic varibles in patients with NPC

Variable	Subset	Hazard ratio (95% CI)	*P*
Univariate analysis (n = 220)			

LATS2 expression	High *vs. *Low	1.978(1.207-3.241)	0.007
Age	< 46 *vs. *≥46	1.563(0.957-2.552)	0.075
Gender	Male *vs. *Female	0.380(0.193-0.746)	0.005
Clinical stage	I, II *vs. *III, IV	1.717(0.932-3.165)	0.083
T-stage	T1+T2 *vs. *T3+T4	1.477(0.889-2.457)	0.132
N-stage	N0 *vs. *N1+N2+N3	0.888(0.525-1.502)	0.658
Histological type (WHO)	II *vs. *III	1.064(0.624-1.815)	0.820
Recurrence	No *vs. *Yes	1.468(0.891-2.418)	0.132
Metastasis	No *vs. *Yes	2.578(1.424-4.665)	0.002

Multivariate analysis (n = 220)			

LATS2 expression	High *vs. *Low	1.714(1.033-2.843)	0.037
Gender	Male *vs. *Female	0.437(0.219-0.874)	0.019
Metastasis	No *vs. *Yes	2.677(1.424-5.031)	0.002

Median age is 46 years

### Methylation analysis of LATS2 in NPC tumors

**Figure 3:**
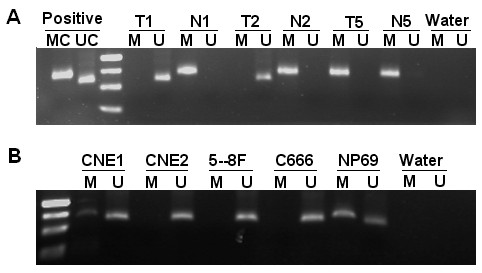
**Methylation-specific PCR analysis of the LATS2 promoter**. Mehtylation of the LATS2 promoter was detected in NPC tumor tissues (A) and NPC cell lines (B). MC, a positive control for methylated alleles. UC, a positive control for unmethylated alleles. M, methylated. U, unmethylated. The methylation-specific products (148 bp) and unmethylation-specific products (130 bp) were separated on 2.5% agarose gels. Marker, 50 bp size marker. T, NPC tumor tissue. N, chronic inflammation of nasopharyngeal samples

Methylation status of LATS2 was analyzed in 30 NPC tissues and 23 nasopharyngeal chronic inflammation samples. Methylation-specific PCR showed that LATS2 promoter methylation was present in 36.7% (11/30) NPC tumor tissues compared with 23 (100%) chronic nasopharyngeal inflammation epithelium samples. (Fig. 3). These results indicated that there was an increased de-methylation frequency of LATS2 in NPC tumor tissues (*P *< 0.01). In NPC cell lines, LATS2 promoter methylation was detected in CNE1 and NP69.

### LATS2 siRNA1 transfection effectively suppresses LATS2 expression

**Figure 4:**
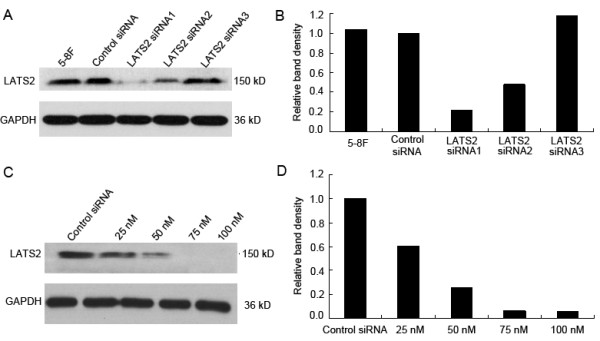
**LATS2 siRNA1 transfection effectively suppresses LATS2 expression**. A, The 5-8F cell line was transfected with three siRNAs (50 nM) to target LATS2 or with control siRNA (50 nM). Cell lysates were generated 72 h post-transfection, followed by immunoblot analysis to determine LATS2 expression; GAPDH was used as the loading control. B, Densitometry analysis revealed that 5-8F cells transfected with LATS2 siRNA1 showed a 78% decrease in LATS2 protein expression compared to cells transfected with control siRNA. C, 5-8F cells were transfected with different concentrations (25 nM, 50 nM, 75 nM and 100 nM) of LATS2 siRNA1 or with control siRNA. Cell lysates were generated at 72 h post-transfection, followed by immunoblot analysis to determine LATS2 expression, GAPDH was used as the loading control. D, Densitometry analysis revealed that 5-8F cells transfected with LATS2 siNRA1 (75 nM and 100 nM) showed a 94% decrease in LATS2 protein expression.

Three siRNAs (50 nM) designed against different regions of LATS2, and a control siRNA (50 nM) were transfected into the 5-8F cell line that showed overexpression of LATS2 based on western blotting results. Cells transfected with LATS2 siRNA1 showed a 78% decrease in LATS2 protein level compared to cells transfected with control siRNA (Fig. 4A and 4B).

Transfection with LATS2 siRNA1 caused a dose dependent decrease in LATS2 protein levels 72 h post-transfection. Cells transfected with LATS2 siRNA1 (75 nM and 100 nM) showed a 94% decrease in LATS2 protein expression compared to cells transfected with control siRNA (Fig. 4C and 4D). Immunohistochemistry analysis also revealed remarkably decreased expression of LATS2 in nuclear of 5-8F cells transfected with 25 nM and 50 nM LATS2 siRNA1, and no expression of LATS2 was observed in 5-8F cells treated with 75 nM LATS2 siRNA1 (Additional file 1). These results suggested that immunostained signals are specific for LATS2 protein, and siRNA mediated LATS2 knockdown in the 5-8F cells resulted the reduction or disappearance of these signals.

### Silencing of LATS2 inhibits growth of 5-8F and CNE2 cells

**Figure 5:**
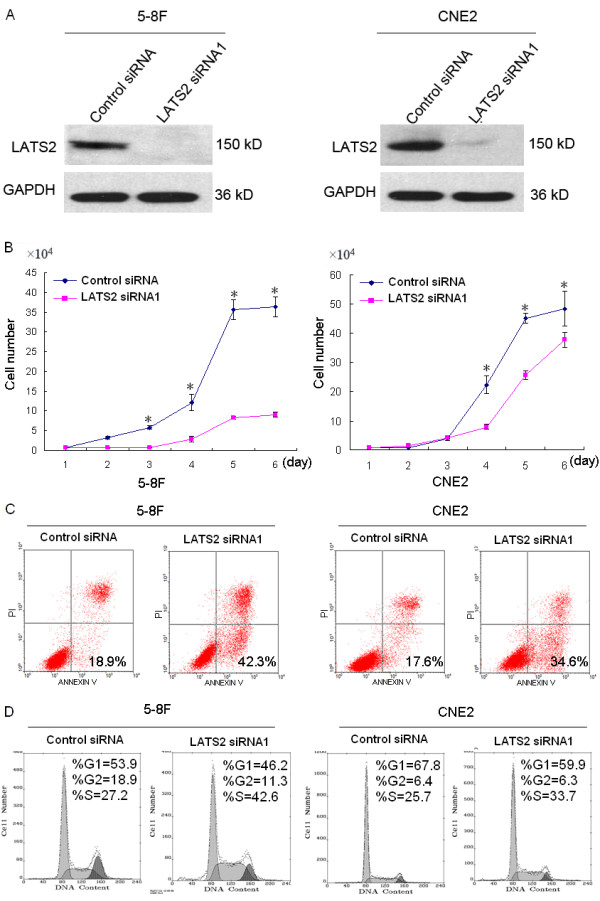
**Suppression of LATS2 expression inhibits growth, induces apoptosis and S-phase increase**. A, 5-8F and CNE2 cell lines were transfected with LATS2 siRNA1 (75 nM) or control siRNA (75 nM). Cell lysates were generated 72 h post-transfection, followed by immoblot analysis to determine LATS2 expression. GAPDH was used as the loading control. B, Growth curve of 5-8F and CNE2 cells upon LATS2 silencing. Results represent the means ± SD (n = 3).*p < 0.05. C, Flow cytometry analysis of apoptosis by using AnnexinV FITC and PI double staining 72 h after transfection. The percentages of apoptotic 5-8F cells transfected with LATS2 siRNA1 and control siRNA were 42.3% and 18.9%, respectively. In CNE2 cells, the percentages of apoptosis in cells transfected with LATS2 siRNA1 and control siRNA were 34.6% and 17.6%. D. Cell cycle distribution was monitored by flow cytometry. The percentages of cells in S-phase in 5-8F cells transfected with LATS2 siRNA1 and control siRNA were 42.6% and 27.2%, respectively. In CNE2 cells, the percentages of S-phase cells among those transfected with LATS2 siRNA1 and control siRNA were 33.7% and 25.7%, respectively.

To assess the effect of LATS2 siRNA1 on cell growth and proliferation in NPC cell lines, 5-8F and CNE2 cells were transiently transfected with LATS2 siRNA1 (75 nM) and control siRNA (75 nM). The total cell number was quantified at 24 h intervals up to 6 days. As shown in Fig. 5B, after transfection there was an apparent decline in the cell number in the LATS2 siRNA1 treated group compared with the control siRNA groups. Especially in 5-8F cells, there was significant inhibition of growth (*P *< 0.05) beginning on the third day after transfection.

### Silencing of LATS2 induces apoptosis in 5-8F and CNE2 cells

Cells treated with LATS2 siRNA1 (75 nM) and control siRNA (75 nM) for 72 h were evaluated for the presence of apoptosis by flow cytometry. The percentage of apoptotic cells was examined by Annexin-V and propidium iodide (PI) double staining. The percentages of apoptotic cells in 5-8F cells transfected with LATS2 siRNA1 and control siRNA were 42.3% and 18.9%, respectively. In the CNE2 cell line, the corresponding values were 34.6% and 17.6%. The ratio of apoptotic cells increased in the LATS2 siRNA1 transfected 5-8F and CNE2 cells as compared to control siRNA transfected cells (Fig. 5C). These results suggest that suppression of LATS2 can lead to induction of apoptosis.

### LATS2 silencing induces S-phase cell cycle increase in 5-8F and CNE2 cells

The cell cycle distribution of transfected cells was examined by flow cytometry. In the 5-8F cell line, the percentage of cells in G1, S and G2 phases in control siRNA (75 nM) treated cells was 53.9%, 27.2% and 18.9%, respectively, and in LATS2 siRNA1 (75 nM) treated cells these percentages were 46.2%, 42.6% and 11.3%, respectively (Fig. 5D). The number of LATS2 siRNA1 treated cells in S-phase was significantly increased, reaching 42.6% 72 h after transfection. Similarly, the proportion of S-phase cells was increased to 33.7% in CNE2 cells transfected with LATS2 siRNA1 compared with 25.7% in control siRNA-transfected cells 72 h after culture. These results indicate that LATS2 silencing results in S-phase cell cycle increase in 5-8F and CNE2 cells.

### Overexpression of LATS2 stimulates cell proliferation

**Figure 6:**
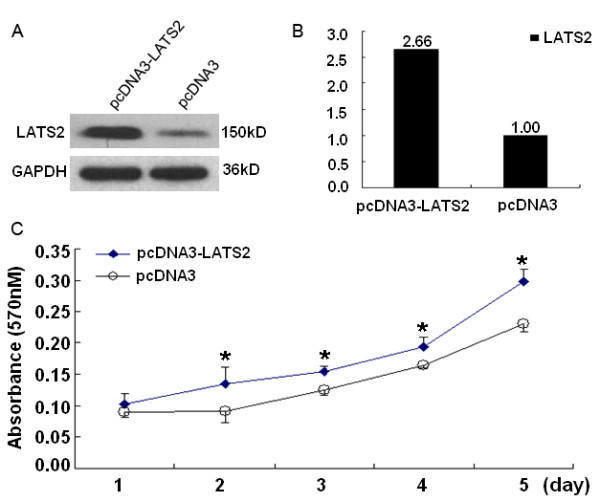
**Overexpression of LATS2 stimulated cell growth**. A, NP69 cells were transfected with pcDNA3-LATS2 or Vector. Cell lysates were generated at 48 h post-transfection, followed by immunoblot analysis to determine LATS2 expression, GAPDH was used as the loading control. B, Densitometry analysis revealed that NP69 cells transfected with pcDNA3-LATS2 showed a 2.66-fold increase in LATS2 protein expression compared to cells transfected with vector. C, MTT assay was used for analysis of the effect of LATS2 on NP69 cell proliferation. Results represent the means ± SD (n = 3).*p < 0.05.

To investigate whether LATS2 overexpression is involved in cell growth, the immortalized nasopharyngeal epithelial cell line NP69 was transfected with pcDNA3-LATS2 and vector. As shown in Fig. 6C, the growth of cells transfected with pcDNA3-LATS2 was evidently increased compared to cells transfected with pcDNA3. These time-effect curves indicated that LATS2 overexpression could stimulate the growth of NP69 cells in vitro.

## Discussion

The Drosophila warts/lats gene is considered to be a tumor suppressor, and down regulation of LATS2 mRNA expression by promoter hypermethylation has been reported to be associated with several malignancies, including breast cancer [26], astrocytoma [28] and acute lymphoblastic leukemia (ALL) [29]. Low expression of LATS2 mRNA has also been reported to be correlated with poor prognosis of patients with ALL [29]. In the present study, LATS2 protein was found to be overexpressed in NPC tumor tissues and NPC cell lines. These results suggest that LATS2 overexpression plays an important role in the development and progression of NPC. Our further retrospective tumor study showed that LATS2 protein was detected in 80.91% (178 of 220 cases) of NPC specimens. It is worth noting that overexpression of LATS2 protein was found to be an indicator of poor prognosis in NPC patients, and LATS2 expression, gender and metastasis were independent predictors of NPC. These results suggest that, as an independent risk factor, LATS2 could serve as a prognostic marker for survival in NPC patients. Furthermore, we found that in 36.7% of cases, the LATS2 promoter is methylated in NPC tumor tissues, while the LATS2 promoter was found to be methylated in all the chronic nasopharyngeal inflammation epithelium samples evaluated (100%). However, this finding needs to be confirmed in a larger series. The present results differ from previously reported data on other malignant cancers, which showed a decrease in LATS2 mRNA levels in tumor tissue [26,28], implying that LATS2 may have different roles in different types of cells and/or cancers.

LATS2 is a component of the Hippo pathway. In mammalian cells, central to the Hippo pathway is consisted of four proteins including Mst1/2, WW45, LATS1/2 and Mob1 [30]. Hippo signaling influences gene expression by regulating transcriptional co-activator YAP (Yes-kinase associated protein), which has a capacity to function either as an oncogene or a promoter of apoptosis [31,32]. LATS1/LATS2 cooperate with the upstream Mst2 kinase to phosphorylate YAP at Ser-127 and inactivate its function as a stimulator of cell detachment [32]. Here we detected the expression of p-YAP in 122 NPC tissues and showed that p-YAP expression was not correlated to the patients survival (P = 0.471, Additional file 2). p-YAP expression was also not correlated with expression of LATS2 (Spearman's correlation coefficient *r *= 0.146, *p *= 0.109). Thus, there may be other factors involved in phosphorylation of YAP in NPC.

There are contradictory reports in the literature regarding the enhancing and/or inhibitory effect of LATS2 on cell survival. LATS2-/- embryos exhibit apoptosis and an arrest in proliferation, whereas LATS2-/- mouse embryonic fibroblasts (MEFs) acquire growth advantages and display a profound defect in contact inhibition of cell growth [20,21]. In lung cancer cell lines (A549 and H1299), overexpression of LATS2 induces apoptosis [18], while it rescues apoptosis induced by nocodazole in the U2OS cell line [19]. Overexpression of LATS2 in Hela cells can cause G2/M arrest [16], while in transformed NIH-3T3 cells, excess LATS2 inhibits G1/S transition [17]. In the present study, the results showed that siRNA-mediated inhibition of LATS2 expression in the human NPC cell line led to inhibition of cell growth, induction of apoptosis and S-phase increase. Overexpression of LATS2 in the NP69 cell line stimulated cell growth. This is consistent with previous studies in LATS2-/- embryos and U2OS cells, but different from results obtained with lung cancer cells and Hela cells. Thus, the effects of LATS2 on cell proliferation or apoptosis seem to be dependent on cell type or cell status. The precise mechanism underlying LATS2 regulated cell survival in NPC needs to be investigated in future studies.

## Conclusions

The present work provides the first evidence of the overexpression of LATS2 in NPC tumor tissues. Overexpression of LATS2 in NPC tumor tissue is an indicator of poor prognosis in NPC patients. SiRNA-mediated downregulation of LATS2 expression results in the inhibition of cell growth, induction of apoptosis and S-phase increase. Overexpression of LATS2 stimulates cell growth. These results suggest that LATS2 might play a role in the tumorigenesis of NPC and might be a potential therapeutic target for NPC treatment.

## Abbreviations

NPC: nasopharyngeal carcinoma; TMA: tissue microarray; MSP: Methylation specific PCR.

## Competing interests

The authors declare that they have no competing interests.

## Authors' contributions

YZ and JYS planned and performed experiments, analyzed data and wrote the manuscript. CFH performed the tissue microarray. JC and LXY reviewed the pathological diagnosis of the NPC samples. YXZ analyzed data and assisted in writing the manuscript. All authors have read and approved the final manuscript

## Pre-publication history

The pre-publication history for this paper can be accessed here:

http://www.biomedcentral.com/1471-2407/10/538/prepub

## Supplementary Material

Additional file 1 **Specificity evaluation of LATS2 by immunohistochemistry**. A, NPC tumor tissues section were incubated in LATS2 primary antibody. B, NPC tumor tssues section were incubated with a non-immunized goat IgG antibody. C, 5-8F cells on the slide were incubated in LATS2 primary antibody. D, 5-8F cells on the slide were incubated with a non-immunized goat IgG antibody. E, LATS2 staining of 5-8F cells transfected with control siRNA. F, LATS2 staining of 5-8F cells transfected with 25 nM LATS2 siRNA1. G, LATS2 staining of 5-8F cells transfected with 50 nM LATS2 siRNA1. H, LATS2 staining of 5-8F cells transfected with 75 nM LATS2 siRNA1. Click here for file

Additional file 2 **Expression of p-YAP in NPC tissue and its association with survival of NPC patients**. A, Expression of p-YAP was determined by immunohistochemistry in 122 NPC tissues. p-YAP was detected in the cytoplasm and nuclear of the NPC tumor cells and nasopharyngeal epithelium cells. B, No significant differences in five-year survival rates were found between low levels of p-YAP expression (n = 73) and high levels of p-YAP expression (n = 49) in NPC patients (*P *= 0.471). C, No significant differences in five-year survival rates were found between low levels of p-YAP expression (n = 19) and high levels of p-YAP expression (n = 12) in NPC patients with early stage disease (stage I - II, P = 0.472). D, No significant differences in five-year survival rates were found between low levels of p-YAP expression (n = 54) and high levels of p-YAP expression (n = 37) in NPC patients with late stage disease (stage III - IV, P = 0.380). Click here for file
