# Supplementary figures and images for: LATS2 is De-methylated and Overexpressed in Nasopharyngeal Carcinoma and Predicts Poor Prognosis
Source: BMC Cancer. 2010 Oct 8;10:538. doi: 10.1186/1471-2407-10-538 (PMC2958949; doi:10.1186/1471-2407-10-538)

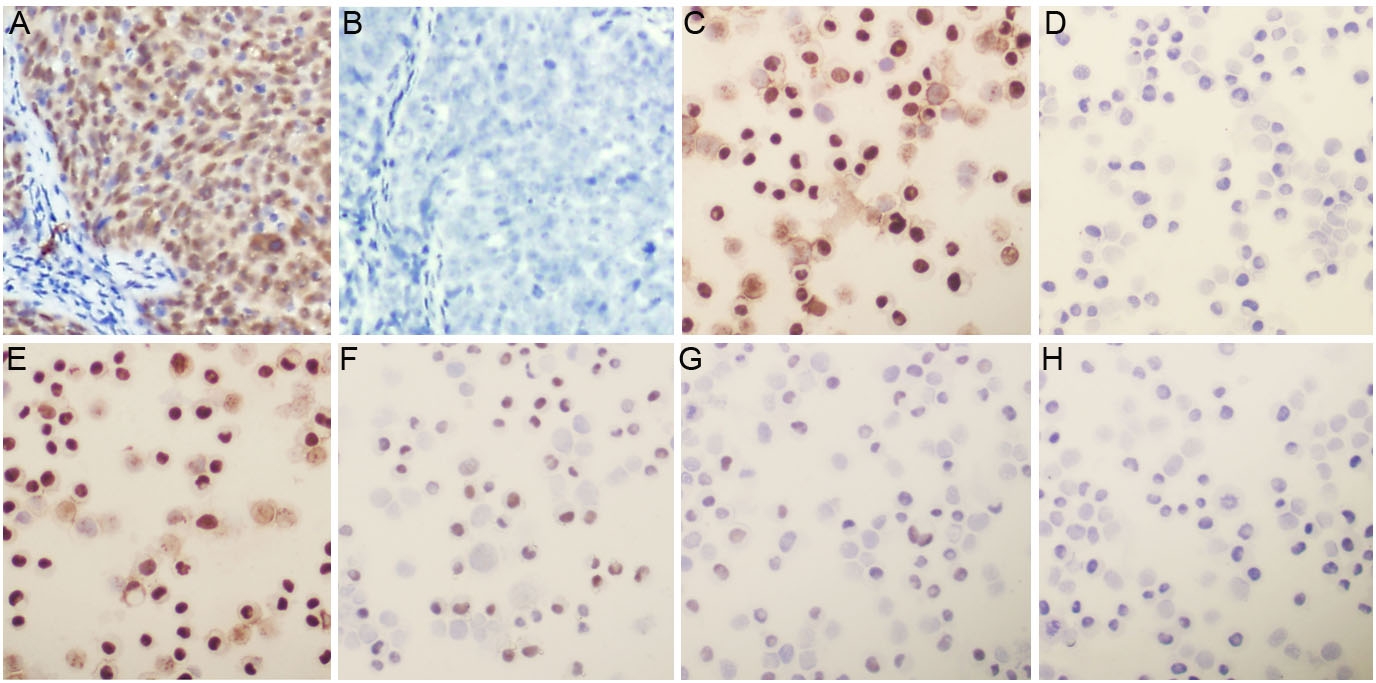

Supplement: Additional file 1 — Specificity evaluation of LATS2 by immunohistochemistry. A, NPC tumor tissues section were incubated in LATS2 primary antibody. B, NPC tumor tssues section were incubated with a non-immunized goat IgG antibody. C, 5-8F cells on the slide were incubated in LATS2 primary antibody. D, 5-8F cells on the slide were incubated with a non-immunized goat IgG antibody. E, LATS2 staining of 5-8F cells transfected with control siRNA. F, LATS2 staining of 5-8F cells transfected with 25 nM LATS2 siRNA1. G, LATS2 staining of 5-8F cells transfected with 50 nM LATS2 siRNA1. H, LATS2 staining of 5-8F cells transfected with 75 nM LATS2 siRNA1. [file 1471-2407-10-538-S1.JPEG]

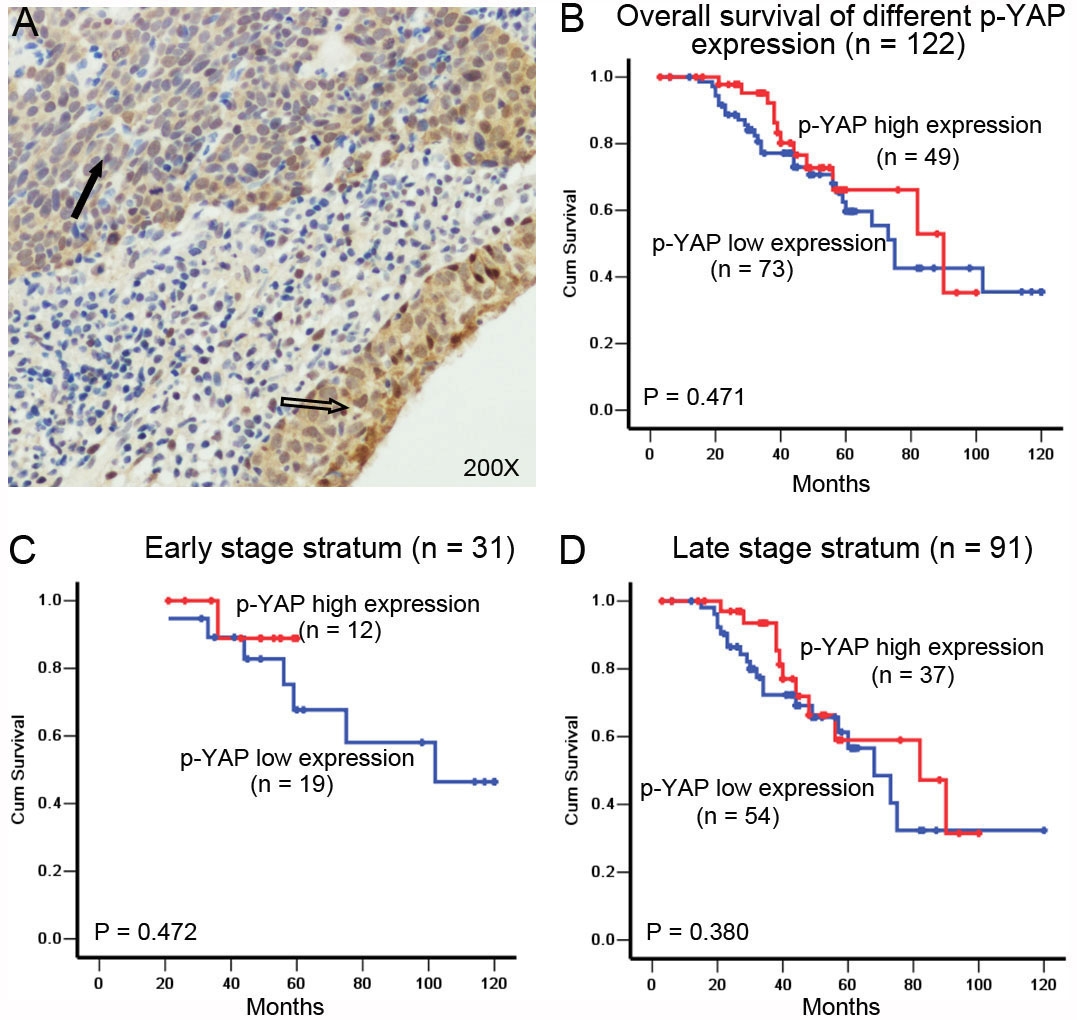

Supplement: Additional file 2 — Expression of p-YAP in NPC tissue and its association with survival of NPC patients. A, Expression of p-YAP was determined by immunohistochemistry in 122 NPC tissues. p-YAP was detected in the cytoplasm and nuclear of the NPC tumor cells and nasopharyngeal epithelium cells. B, No significant differences in five-year survival rates were found between low levels of p-YAP expression (n = 73) and high levels of p-YAP expression (n = 49) in NPC patients (P = 0.471). C, No significant differences in five-year survival rates were found between low levels of p-YAP expression (n = 19) and high levels of p-YAP expression (n = 12) in NPC patients with early stage disease (stage I - II, P = 0.472). D, No significant differences in five-year survival rates were found between low levels of p-YAP expression (n = 54) and high levels of p-YAP expression (n = 37) in NPC patients with late stage disease (stage III - IV, P = 0.380). [file 1471-2407-10-538-S2.JPEG]
